# Biomarkers of Glyco-Metabolic Control in Hemodialysis Patients: Glycated Hemoglobin vs. Glycated Albumin

**DOI:** 10.3390/medicina57070712

**Published:** 2021-07-14

**Authors:** Francesca Gabriela Martino, Marina Vitillo, Massimo Pieri, Giulia Marrone, Fabio Gangeri, Ferruccio Ansali, Mariarita Dessì, Sergio Bernardini, Nicola Di Daniele, Annalisa Noce

**Affiliations:** 1 UOC Clinical Pathology, Hospital San Filippo Neri Laboratory HUB, ASL Roma 1, 00135 Rome, Italy; francescag.martino@aslroma1.it (F.G.M.); marina.vitillo@aslroma1.it (M.V.); 2 Department of Experimental Medicine, Clinical Biochemistry, University of Rome Tor Vergata, 00133 Rome, Italy; massimo.pieri@uniroma2.it (M.P.); mariarita.dessi@uniroma2.it (M.D.); bernards@uniroma2.it (S.B.); 3 UOC of Internal Medicine—Center of Hypertension and Nephrology Unit, Department of Systems Medicine, University of Rome Tor Vergata Via Montpellier, 00133 Rome, Italy; giul.marr@gmail.com (G.M.); didaniele@med.uniroma2.it (N.D.D.); 4 PhD School of Applied Medical, Surgical Sciences, University of Rome Tor Vergata, 00133 Rome, Italy; 5 UOC Nephrology and Dialysis, Santo Spirito Hospital, ASL Roma 1, 00193 Rome, Italy; fabio.gangeri@aslroma1.it; 6 UOC Nephrology and Dialysis, San Paolo Hospital, ASL Roma 4, 00053 Civitavecchia, Italy; ferruccio.ansali@aslroma4.it; 7 UDD Nephrology and Dialysis, Padre Pio Hospital, ASL Roma 4, 00062 Bracciano, Italy

**Keywords:** diabetes mellitus, hemodialysis, glycated hemoglobin, glycated albumin, glyco-metabolic biomarkers

## Abstract

*Background and Objectives:* Glycated hemoglobin (HbA1c) dosage is considered the gold standard in glycol-metabolic monitoring, but it presents limits, which can underestimate the glycemia trend. In this regard, it was introduced the glycated albumin (GA). The aim of the study is to verify the predictivity of the GA compared to HbA1c in identifying glyco-metabolic alterations in non-diabetic and diabetic hemodialysis (HD) patients. *Materials and Methods:* For this purpose, we conducted a multicenter study involving one analysis laboratory and six dialysis centers in the Lazio region (Rome, Italy). Both diabetic and non-diabetic HD patients represent the study population, and the protocol included five time points. *Results:* The analyzed data highlighted the ability of GA to predict changes in glycemic metabolism in HD patients, and GA values are not significantly influenced, like HbA1c, by dialysis therapy itself and by comorbidities of the uremic state, such as normochromic and normocytic anemia. Thus, GA seems to reflect early glyco-metabolic alterations, both in patients with a previous diagnosis of diabetes and in subjects without diabetes mellitus. As part of this study, we analyzed two HD patients (one diabetic and one non-diabetic) in which GA was more predictive of glycol-metabolic alterations compared to HbA1c. Our study confirms the need to compare classical biomarkers used for the monitoring of glyco-metabolic alterations with new ones, likely more reliable and effective in specific subgroups of patients in which the classic biomarkers can be influenced by the preexisting pathological conditions. *Conclusions*: In conclusion, our evidence highlights that in uremic patients, GA shows a better ability to predict glyco-metabolic alterations allowing both an earlier diagnosis of DM and a prompt modulation of the hypoglycemic therapy, thus improving the clinical management of these patients.

## 1. Introduction

In 2019, the estimated prevalence of chronic kidney disease (CKD) in the world was 13.4% [1]. This prevalence increases up to 15–30% in the elderly compared to the adult population and exceeds 50% in subjects affected by cardiovascular (CV) and metabolic diseases [2,3,4]. Among the causes of CKD, the most common are nephroangiosclerosis and diabetic kidney disease (DKD) [5]. These two pathological conditions are linked to high blood pressure and long-lasting diabetes mellitus (DM), and they represent almost half of all the causes of CKD [6]. As reported by Duru et al., the prevalence of DKD in the USA (2017) was approximately 25% in diabetic patients, and it was estimated that in Europe, the prevalence of CKD in diabetic patients for the period 2012–2025 would increase significantly up to 18% in men and over 21% in women [7,8,9,10]. The onset of DKD is related to a lot of risk factors: (i) predisposing ones such as age, gender, race–ethnicity, and family history, (ii) triggering ones, such as hyperglycemia and acute kidney injury, and (iii) progression factors, such as arterial hypertension, dietetic habits, and excessive body weight [11,12,13].

Currently, the two major epicenters of the DM are represented by China and India, and they are characterized, at the time of DM diagnosis, by lower body mass index (BMI) and younger age, compared to Western Countries [9,14]. The DM prevalence increase is mainly attributable to the rampant diffusion of overweight and obesity in developed countries and to the easier access to food sources in developing countries [15]. Therefore, it is necessary to set up preventive strategies and to test biomarkers that allow an early diagnosis to stem this phenomenon [16,17,18].

The term DM refers to a group of metabolic disorders associated with hyperglycemia. According to the American Diabetes Association (ADA), the diagnosis of DM is made on the basis of the presence of clinical symptoms, such as hyperglycemia or hyperglycemic crisis, a random blood glucose > 200 mg/dL or the glycated hemoglobin (HbA1c) ≥ 48 mmol/mol (6.5%), and fasting plasma glucose (FPG) ≥ 126 mg/dL (7.0 mmol/L) [19].

Glycated proteins, especially glycated hemoglobin (HbA1c), are widely used for the long-term monitoring of glycemia values [20]. HbA1c dosage is commonly used in clinical practice as it reflects the average plasma glucose values over the past 90–120 days [20,21,22]. Although it is considered the gold standard for glycol-metabolic monitoring, HbA1c presents some limits. In fact, any condition that is able to induce a reduction in the survival of the erythrocytes or to reduce the half-lifespan of the same (such as acute hemorrhages, hemolytic anemias, and CKD) can underestimate the HbA1c values. In particular, in CKD patients, red blood cells lifespan is reduced because of uremic toxins accumulation and decreased glucose-6-phosphate dehydrogenase (G6PD) activity [23,24,25,26].

The main cause of anemia in CKD patients is represented by the reduction in erythropoietin (EPO) production [27]. In addition, iron deficiency anemia, characterized by Hb levels < 13 g/dL in men and Hb < 12 g/dL in women associated with low serum iron <7.1 µg/L, low serum ferritin <30 ng/L, low transferrin saturation percentage (<15%), and high total iron-binding capacity > 13.1 µmol/L, could overestimate HbA1c values [28,29,30,31]. Moreover, the HbA1c values may be falsely increased or decreased in the case of hemoglobinopathies [32]. DM is a further risk factor for anemia: 10% of DM patients with preserved renal function are anemic [33]. In a diabetic patient, the appearance of anemia is favoured by peripheral neuropathy, which could compromise the detection of O_2_ necessary for the production of EPO. In the DKD patients, anemia is more frequent with early-onset compared to non-diabetic CKD patients [34].

In diabetic chronic maintenance HD patients, glycemic control plays a fundamental role in reducing the progression of complications related to diabetes and in increasing survival. These subjects have more frequent episodes of hypoglycemia both the intradialytic period, due to the glucose removal during the dialysis session, and in the interdialytic interval due to the reduced renal clearance of insulin, which determines an extension of its half-life, and for reduced renal gluconeogenesis [35,36,37].

The glycemic monitoring of the HD patient through HbA1c is therefore significantly influenced by different variables that could both overestimate and underestimate it, reducing its reliability. Considering the high prevalence of these two pathological conditions in the same patients, the need of a biomarker capable of overcoming the limits shown by HbA1c appears evident. In this regard, it was introduced the glycated albumin (GA). High GA levels can induce irreversible damage in the different target organs of DM (such as the CV system, the kidney, the retina, and the nervous system). Compared to HbA1c, which is a long-term glycemic indicator (about 120 days), GA is a medium-term biomarker (about 20 days) since it reflects the average life of albumin. This means that, theoretically, GA can immediately indicate both an improvement and a worsening of the glycemic compensation, and it is, therefore, useful in all those conditions that require a short-term control of average glycemic values.

The aim of the study is to verify the predictivity of GA compared to HbA1c in identifying glyco-metabolic alterations in non-diabetic and diabetic HD patients.

For this purpose, we conducted a multicenter study involving the San Filippo Neri Hospital in Rome, the University Hospital Policlinico Tor Vergata in Rome, the Santo Spirito Hospital in Rome, the San Paolo Hospital in Civitavecchia, the Padre Pio Hospital in Bracciano, the San Feliciano Dialysis Center in Rome, and the Dialysis Center of the ARS Medica in Rome.

## 2. Materials and Methods

Both diabetic and non-diabetic HD patients represent the study population. The inclusion criteria for diabetic patients were chronic HD for at least 3 months; DM type 1 or 2; both sexes; age over 18 years; BMI between 20 and 30 kg/m^2^. For this group of patients, the exclusion criteria were the presence of hyperthyroidism and albumin <3 g/dL. For non-diabetic patients, the criteria were the same as in the previous group, excluding the presence of DM. Patients signed the informed consent as per the protocol accepted by the Independent Ethics Committee of the different hospitals that participated in this multicentric study (Ethical Committee Lazio 1 register number 1250/18 of 20 June 2018, Ethical Committee Tor Vergata register number 209/18 of 19 December 2018).

GA and HbA1c detection were performed every 30 days for three months (T0, T1, T2, and T3), and the final sampling was made after 6 months (T4). Basal dosage was sampled on plasma-EDTA for HbA1c and on whole blood for GA. HbA1c was obtained through the use of Capillarys Flex Piercing (SEBIA, Lisses, France), and GA was performed using the quantILab glycated albumin kit (Instrumentation Laboratory, Ascoli Piceno, Italy) implemented on the COBAS c702 Module (ROCHE Diagnostics, Mannheim, Germany). Detections were all performed at the UOC Clinical Pathology, Hospital San Filippo Neri, Laboratory HUB of ASL Roma 1.

**Figure 1:**
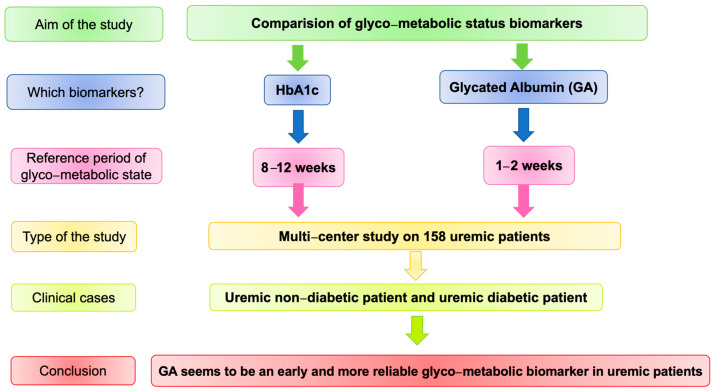
Design of the study. Abbreviation: HbA1c, glycated hemoglobin.

The study design is summarized in Figure 1. In particular, the study was conducted in 5 time points: T0, T1, T2, T3 (monthly), and T4 (after 6 months). At T0, after the acquisition of the patient’s signed informed consent, a medical history was collected together with some anthropometric parameters, such as weight, height, and BMI. The patients involved in the study underwent dialysis three times a week, either in the morning or in the afternoon shift.

Blood samples were then collected in order to analyze various blood chemistry parameters, such as GA, HbA1c, glycemia, albumin, and hemoglobin.

The laboratory tests were carried out at the Clinical Pathology of the San Filippo Neri Hospital. The samples were stored at −80 °C until the time of analysis.

All the samples used in the investigation were kept anonymous by means of double-coded identification codes. The data were collected at the Clinical Pathology of the San Filippo Neri Hospital—ASL Roma 1—and analyzed anonymously in compliance with current legislation on the processing of personal data (Legislative Decree 196/2003). The samples were kept for about 12 months, i.e., the duration of the study.

All statistical analyses were performed using SPSS 17.0 software (IBM Corporation, New York, NY, USA) to determine mean ± standard deviations and percentages between the different groups. An analysis of variance (ANOVA) with Bonferroni was used as a post hoc test to highlight any statistical difference between the different groups. The receiver operating characteristic (ROC) curves were calculated to determine the sensitivity and specificity of HbA1c and GA to the respective cut-offs that allowed the diagnosis of DM. Values of *p* < 0.05 were considered significant.

## 3. Results

**Figure 2:**
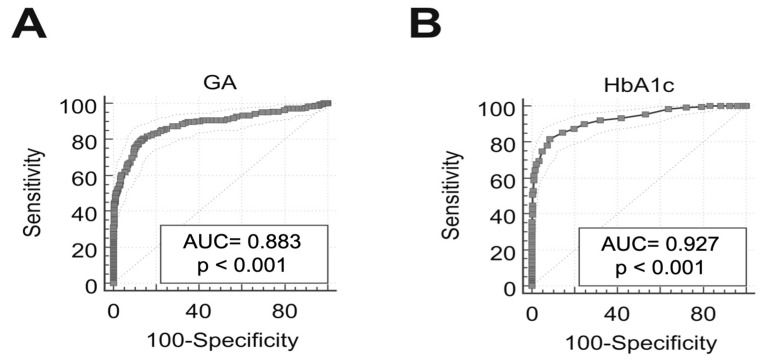
The ROC curves of GA (panel **A**) and HbA1c (panel **B**). Abbreviations: AUC, area under the curve; GA, glycated albumin; HbA1c, glycated hemoglobin; *p*, *p*-value; ROC, receiver operating characteristic.

**Figure 3:**
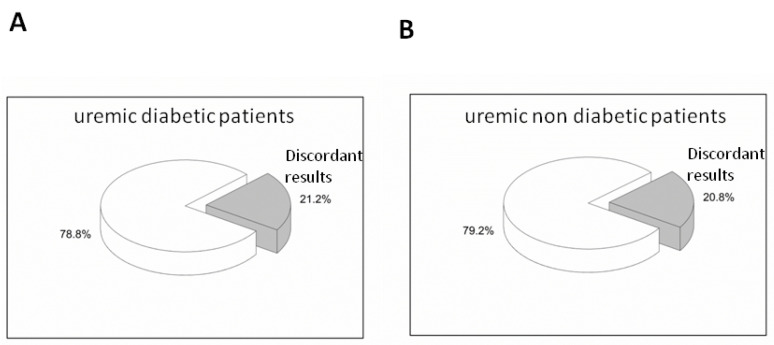
Discrepancies between the GA and HbA1c values in uremic diabetic (panel **A**) and uremic non-diabetic (panel **B**) patients. Abbreviations; GA, glycated albumin; HbA1c, glycated hemoglobin.

**Figure 4:**
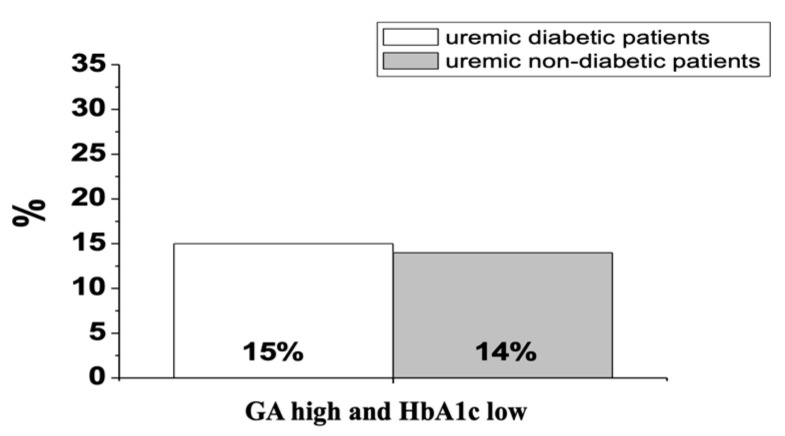
Percentage of discordant values between GA and HbA1c in Group 1 (uremic diabetic patients) and Group 2 (uremic non-diabetic patients). Abbreviations: GA, glycated albumin; HbA1c, glycated hemoglobin.

This study was conducted on 160 HD patients. The epidemiological findings of the enrolled population were: 102 men (63.8%) and 58 women (36.2%); 60 diabetics (37.5%), 98 non-diabetics (61.3%) and 2 with impaired glucose tolerance (IGT; 1.2%). These data are summarized in Table 1. The obtained GA and HbA1c values showed a significant correlation between the two variables in the HD population (R = 0.78, *p*-value < 0.0001). This correlation was also more evident within the Group 1, uremic diabetic patients, (R = 0.71; *p*-value < 0.0001) but not in Group 2, non-diabetics uremic patients, (R = 0.20; *p* > 0.05). The statistical analysis of these data allowed us to identify the sensitivity and specificity of HbA1c and GA. The latter for the cut-off of 14.5% had a sensitivity and specificity of 84.77 and 77.95, respectively. Instead, the HbA1c for the cut-off of 48 mmol/mol presented a sensitivity and specificity of 39.51 and 99.55, respectively. The ROC curves of GA and HbA1c (Figure 2) showed an area under the curve (AUC) of 0.883 for the first one and 0.927 for the second one. For both parameters, the *p*-value was <0.001. All the 160 uremic patients participating in this study were monitored for the HbA1c and GA at five different time points with a total of 805 determinations of both glyco-metabolic biomarkers. Analysis of the data obtained showed some discrepancies between the GA and HbA1c values. In particular, as regards Group 1, the discrepancies found were equal to 21.2% (Figure 3). Among these, 61.2% were characterized by GA values above the cut-off already from T0 and by HbA1c values in the normal range at T0 with a progressive increase in subsequent measurement times of the study. In Group 2, we found 20.8% of the discordant values (Figure 3). None of the HD non-diabetic uremic patients had normal GA values associated with HbA1c values above the cut-off at all time points of measurement. In Group 1, we found that 15% of the discordant values were due to high GA and low HbA1c values in all measurement time points, while in Group 2, discordant concentrations between the two biomarkers were detected in 14% of patients (Figure 4). Finally, two clinical cases were selected, whose GA values were >14.5% (namely pathological value) since T0, while the HbA1c values were normal. The features of the two selected HD patients, one diabetic and one non-diabetic, are listed in Table 2 and Table 3. Moreover, GA values were in agreement with the basal blood glucose measured at the different time points of the study. In addition, in the HD non-diabetic selected patient, we found the same trend of the two examined biomarkers.

## 4. Discussion

DM represents an important CV and all-cause mortality risk factor [38,39]. Recent studies have shown that an optimal glyco-metabolic control, combined with a periodic follow-up of renal function, is essential to prevent or limit the comorbidities related to due DM, above all DKD. Good glycemic control plays a primary role in preventing the onset of micro and macrovascular complications, typical of DM [40,41].

HD patients, due to the observed changes in glucose metabolism and hematopoiesis, have lower reliability in sampling HbA1c, which currently is the gold standard for the monitoring of glyco-metabolic metabolism. As previously discussed, a series of pathological mechanisms can influence the reliability of HbA1c in uremic patients [42,43,44,45,46]. For this reason, we hypothesized that GA should represent a new possible and more reliable glyco-metabolic biomarker in HD patients to be placed alongside the currently used traditional biomarkers.

Literature data highlighted that DKD increases in a proportional manner with age, as the decline of renal function is physiologically related to aging. In fact, with aging, the kidney undergoes functional and structural changes, and this phenomenon is amplified in the presence of DM. The risk factors related to DM that contribute to premature aging of the kidney and the cardiovascular system are the accumulation of advanced glycation products, increased oxidative stress, chronic low-grade inflammatory state, and accelerated atherosclerosis [47,48]. Moreover, the average age of patients in renal replacement therapy has been characterized by its progressive increase over the years, passing from a mean age of 55 years in 2002, to a mean age of 62 years in 2017 [49]. In our population, the mean age was 64.1 ± 12.6 years, according to literature data [50]. A possible explanation for this phenomenon is related to the lengthening of the average life span of the general population, to an easier access to treatment, and to a better clinical management of diabetic and CKD patients.

Anyway, our study population was characterized by a higher percentage of men compared to women, according to literature data [51,52]. The higher prevalence of CKD observed in men is likely to be related to the higher prevalence in males of the risk factors for nephropathy, such as obesity, DM, metabolic syndrome, and arterial hypertension [53].

Our data allowed us to determine the sensitivity and specificity of GA and HbA1c according to their respective cut-offs. Therefore, evaluating the two ROC curves, it is clear that in HD, GA presented higher sensitivity than HbA1c (84.77 vs. 39.51), and it would seem to have a better predictive capacity in identifying new cases of DM. On the contrary, HbA1c would seem to have a greater ability to identify non-diabetic HD subjects due to its sensitivity (77.95 vs. 99.55). 

In the uremic diabetic and non-diabetic patients, the discrepancies found were 21.2% and 20.8%, respectively. They were characterized by GA values above the cut-off already from T0 and by HbA1c values in the normal range at T0 with a progressive increase in subsequent measurement times. This seems to confirm the predictive value of GA. In fact, most of the discordant cases were due to high GA and low HbA1c values in all measurement times, and they were 15% in non-diabetic uremic patients and 14% in uremic diabetic patients.

Our data agree with a previous study conducted by Mo et al. on 953 diabetic patients. In fact, these authors demonstrated that GA was positively correlated with the HbA1c values in all enrolled patients [54]. Therefore, GA should be used in association with HbA1c to monitor glyco-metabolic status in HD patients. 

Besides our evidence highlighted that in uremic patients, GA showed a better ability to predict the glyco-metabolic alterations allowing both an earlier diagnosis of DM and a timely modulation of low-glycemic therapy, thus improving the clinical management of these patients.

Once CKD has been established, it is important to make an early diagnosis of DM with additional biomarkers that are able to assess the glyco-metabolic profile of the HD patient. The monitoring is often difficult for the physician, and if it is not well performed, it the onset of comorbidities typical of DM, worsening the quality of life. In addition, the association between the trend over time of the two biomarkers and the causes of mortality should be investigated in a larger clinical observational study [55].

Despite the evidence that emerged from our study, as well as from the previously mentioned studies, GA cannot yet be officially recognized as a standardized glyco-metabolic biomarker in HD patients as further investigations with a major cohort and with a longer observational time are needed to validate its routine use.

Its future validation could lead to the improvement in glyco-metabolic control of the diabetic HD patient, simplifying the clinical monitoring, which is often difficult to interpret for the physicians. 

## 5. Conclusions

The evidence that emerged from this study were various: the high specificity of both GA and HbA1c, the greater sensitivity of GA compared to HbA1c in identifying new cases of DM in HD patients, and the greater predictive capacity of GA in detecting early glyco-metabolic alterations.

## Figures and Tables

**Table 1:** Epidemiological data of the 160 hemodialysis patients enrolled in the study. Abbreviations: BMI, body mass index; IGT, impaired glucose tolerance; SD, standard deviation.

	Total	Men	Women
N (%)	160	102 (63.8)	58 (36.2)
Age; mean ± SD, years	64.1 ± 12.6	64 ± 13	64 ± 11
BMI; mean ± SD, kg/m^2^	24.8 ± 3.5	25 ± 3.5	24.4 ± 3.4
Diabetic uremic patients; N (%)	60 (37.5)	46 (77)	14 (23)
Non-diabetic uremic patients; N (%)	98 (61.3)	55 (56)	43 (44)
IGT; N (%)	2 (1.2)	1 (50)	1 (50)

**Table 2:** Features of the two patients, one diabetic and one non-diabetic. Abbreviations: BMI, body mass index; EPO, erythropoietin. * Insulin therapy: three times a day, 6 international unit (IU) of rapid-acting insulin (hour 8:00 a.m., 1:00 p.m. and 7:00 p.m.) and once a day, 4 IU of long-acting insulin (hour 10.00 p.m.).

Patient	Diabetic *	Non-Diabetic
Sex	M	M
Age (years)	75	72
Type of dialysis	Convective technique	Diffusive technique
Type and dosage of EPO	Epoetin-α 4000 IU × 2/week	No therapy
BMI (kg/m^2^)	23.8	21.4

**Table 3:** Biomarkers of glyco-metabolic status in each time point of the study. Abbreviations: A, albumin; GA, glycated albumin; HbA1c, glycated hemoglobin.

Patient	Diabetic	Non-Diabetic	Normal Range Values
Glycemia mg/dL (T0)	99	137	80–100 mg/dL
Glycemia mg/dL (T1)	151	160	
Glycemia mg/dL (T2)	161	120	
Glycemia mg/dL (T3)	95	105	
Glycemia mg/dL (T4)	80	126	
HbA1c mmol/mol/Hb g/dL (T0)	34/10.5	33/10.7	HbA1c: <38 mmol/mol (Normal) 39–47 mmol/mol (Pre-diabetes) >48 mmol/mol (Diabetes)
HbA1c mmol/mol/Hb g/dL (T1)	38/9.6	35/10.6	
HbA1c mmol/mol/Hb g/dL (T2)	36/9.8	35/10.8	
HbA1c mmol/mol/Hb g/dL (T3)	30/10.2	35/11.0	
HbA1c mmol/mol/Hb g/dL (T4)	35/11.1	36/11.3	
GA %/A g/dL (T0)	14.3/4.5	17.2/5.8	GA ≤ 15%
GA %/A g/dL (T1)	16.5/4.8	16.0/5.7	
GA %/A g/dL (T2)	16.3/5.0	16.4/5.8	
GA %/A g/dL (T3)	14.6/5.0	16.4/5.3	
GA %/A g/dL (T4)	14.9/5.1	15.4/5.5	

## Data Availability

Data available on request due to privacy restriction. The data presented in this study are available on request from the corresponding author.
